# Supplementary figures and images for: Potentially inappropriate medication and attitudes of older adults towards deprescribing
Source: PLoS One. 2020 Oct 26;15(10):e0240463. doi: 10.1371/journal.pone.0240463 (PMC7588126; doi:10.1371/journal.pone.0240463)

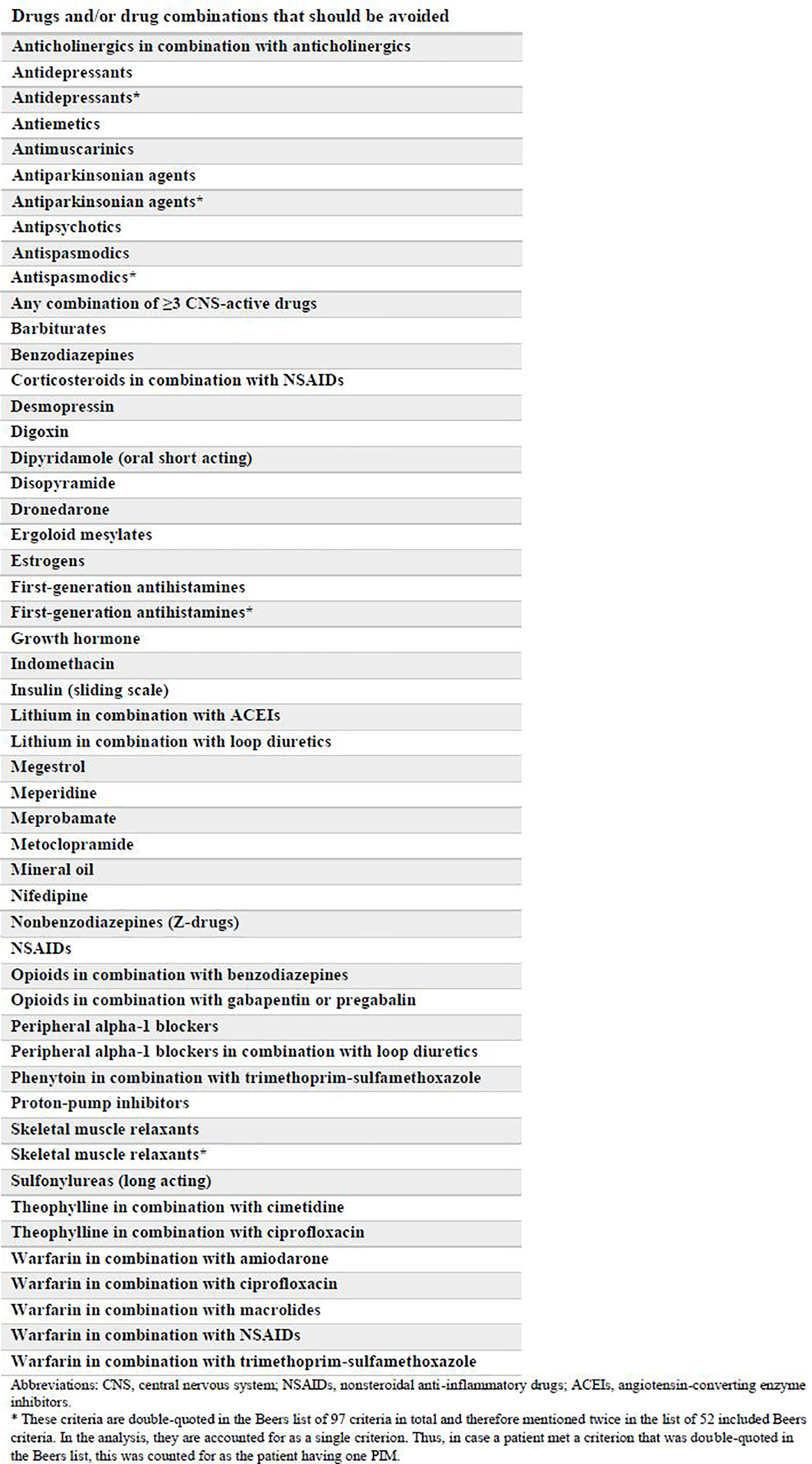

Supplement: S1 Appendix — (TIF) [file pone.0240463.s002.tif]

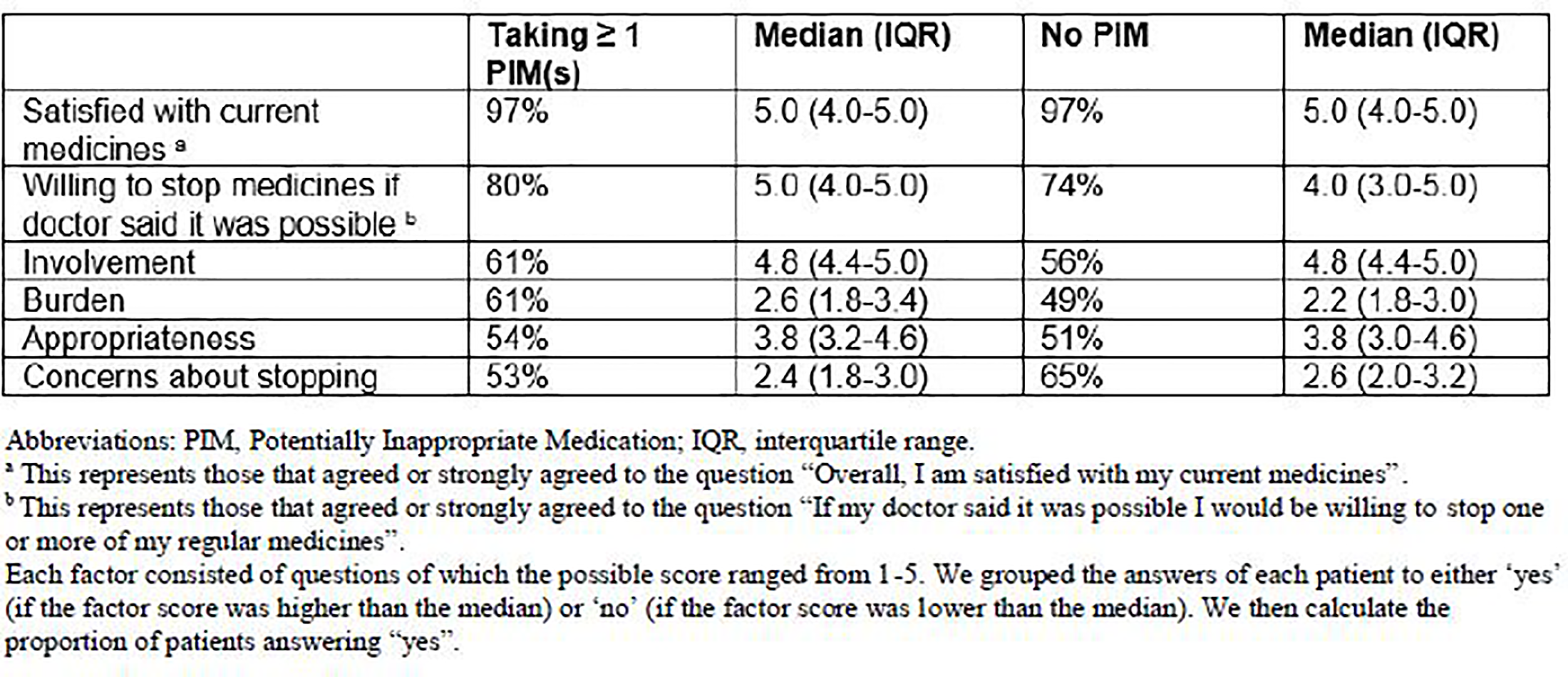

Supplement: S2 Appendix — (TIF) [file pone.0240463.s003.tif]
