# Supplementary material for: Potentially inappropriate medication and attitudes of older adults towards deprescribing
Source: PLoS One. 2020 Oct 26;15(10):e0240463. doi: 10.1371/journal.pone.0240463 (PMC7588126; doi:10.1371/journal.pone.0240463)
Supplement: Attachment — Submitted filename: Response to Reviewers.docx [file pone.0240463.s004.docx]

Recipient

Christine Leong, Pharm. D.

Academic Editor

PLOS ONE

**Revision of manuscript ID:** PONE-D-20-16955

Dear Dr Leong, dear PLOS ONE Editorial Board,

We were invited to submit a revised version of our manuscript:

**“Potentially inappropriate medication use and attitudes of older adults towards deprescribing”**

We accepted the opportunity to revise and resubmit a revised version of this manuscript and we would like to thank you for this opportunity.

We wrote a response to the reviewer’s comments that is attached below, answering every comment point-by-point. With that, we hope to have sufficiently answered and implemented all questions, suggestions and comments.

We dearly hope to have made the necessary adaptations for convincing you to accept our revised submission. Again, we want to express our gratitude for the opportunity to improve our manuscript with the relevant and valuable input from the reviewer.

Yours sincerely,

Prof. Sven Streit, MD, MSc, PhD

Professor in Primary Care, Head of Interprofessional Primary Care
Institute of Primary Health Care Bern (BIHAM)

University of Bern, Mittelstrasse 43
3012 Bern, Switzerland
Tel +41 31 631 58 75
Email: sven.streit@biham.unibe.ch

**Point-by-point response to reviewers' comments:**

1. If the authors have adequately addressed your comments raised in a previous round of review and you feel that this manuscript is now acceptable for publication, you may indicate that here to bypass the “Comments to the Author” section, enter your conflict of interest statement in the “Confidential to Editor” section, and submit your "Accept" recommendation.

Reviewer #2: (No Response)

*Response: We would like to thank the reviewer for his positive feedback on our first revision. We have address the additional comments in the point-by-point response below.*

2. Is the manuscript technically sound, and do the data support the conclusions?

The manuscript must describe a technically sound piece of scientific research with data that supports the conclusions. Experiments must have been conducted rigorously, with appropriate controls, replication, and sample sizes. The conclusions must be drawn appropriately based on the data presented.

Reviewer #2: Yes

*Response: Thank you for this comment. We are happy to read that we addressed the comments in the first revision adequately.*

3. Has the statistical analysis been performed appropriately and rigorously?

Reviewer #2: Yes

*Response: We would like to thank the reviewer for this assessment.*

4. Have the authors made all data underlying the findings in their manuscript fully available?

The PLOS Data policy requires authors to make all data underlying the findings described in their manuscript fully available without restriction, with rare exception (please refer to the Data Availability Statement in the manuscript PDF file). The data should be provided as part of the manuscript or its supporting information, or deposited to a public repository. For example, in addition to summary statistics, the data points behind means, medians and variance measures should be available. If there are restrictions on publicly sharing data—e.g. participant privacy or use of data from a third party—those must be specified.

Reviewer #2: Yes

*Response: Thank you.*

5. Is the manuscript presented in an intelligible fashion and written in standard English?

PLOS ONE does not copyedit accepted manuscripts, so the language in submitted articles must be clear, correct, and unambiguous. Any typographical or grammatical errors should be corrected at revision, so please note any specific errors here.

Reviewer #2: Yes

*Response: Thank you.*

6. Review Comments to the Author

Please use the space provided to explain your answers to the questions above. You may also include additional comments for the author, including concerns about dual publication, research ethics, or publication ethics. (Please upload your review as an attachment if it exceeds 20,000 characters)

Reviewer #2: Thank you for the responses to my review. I feel they have addressed the majority of issues.

*Response: We would like to thank the review for this assessment. The new comments are addressed in the point-by-point response below.*

1. Thank you for the clarification that each GP was in a different practice/clinic. This should be added to the Methods section.

*Response: Thank you. We have added this information to the methods section (p. 5, l. 2). The text now reads: “All of them were located in different GP offices.”*

2. Regarding screening, the newly inserted sentence says GPs were instructed to consecutively screen 5 eligible patients. Should this be "to consecutively screen eligible patients and recruit 5 participants, reporting the number of patients screened..."?

*Response: Thank you for highlighting this error. We have replaced the sentence as suggested. The text now reads: “GPs were instructed to consecutively screen eligible patients and recruit 5 participants, reporting the number of patients screened, to reduce the risk of selection bias” (p. 6, l. 6-7).*

3. The new legend that was intended for Figure 1 appears to have been inserted below the Figure 2 title in error.

*Response: Thank you. We have moved the figure legend.*

4. Two revisions to the manuscript mentioned in the response letter have not actually been included in the manuscript (they have been inserted and then deleted in the tracked changes version). These related to hypothesising patients using PIMs were more likely to experience side effects in the Introduction, and that the study may not have been powered for examining individual questions in the Limitations. I feel these are both important and warrant inclusion.

Response: Thank you for highlighting this shortcoming. We have inserted these two revision in the manuscript text.

1) “We hypothesized that patients who use PIMs experience more side effects than patients who do not use any PIMs, which in turn might affect their willingness to deprescribe” (p. 4, l. 15-17). (this sentence was adapted based on the comment below)

2) “Furthermore, very few of the individual factors had evidence to support a relationship with the use of PIMs, which stems from the fact that the study might not have been sufficiently powered to detect such differences.” (p. 11, l. 11-13)

5. The proposed addition relating to the hypothesis may warrant rephrasing as it is contradictory as is : "We hypothesized that patients who use PIMs, irrespective of whether the medication they use are potentially inappropriate, experience...". Should the part beginning irrespective refer to the number of medications they use, or simply be removed?

Response: Thank you. We have removed the part “irrespective of whether the medication they use are potentially inappropriate” from the manuscript text.

7. PLOS authors have the option to publish the peer review history of their article (what does this mean?). If published, this will include your full peer review and any attached files.

Response: I agree
